# Supplementary material for: DMRforPairs: identifying Differentially Methylated Regions between unique samples using array based methylation profiles
Source: BMC Bioinformatics. 2014 May 15;15:141. doi: 10.1186/1471-2105-15-141 (PMC4046028; doi:10.1186/1471-2105-15-141)
Supplement: Additional file 2 — DMRforPairs output for the comparison of A431-MCF7 and NA17018-NA17105. Please start from the HTML files in each folder. Available via the BMC Bioinformatics website. [file 1471-2105-15-141-S2.zip › 1394847754114233_MOESM2_ESM/A431_MCF7/figures/30682.pdf]

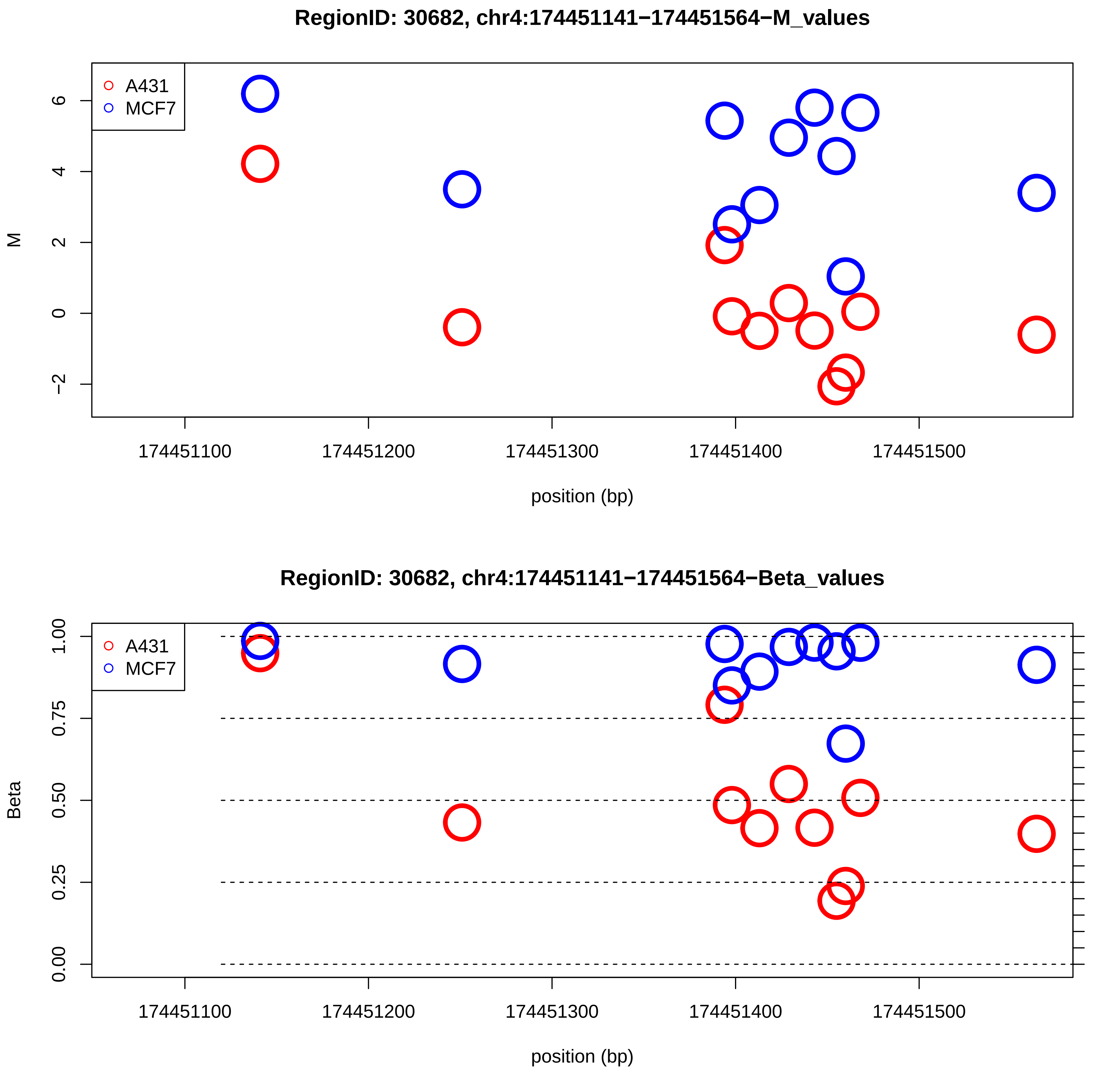

RegionID: 30682, chr4:174451141−174451564−M_values
A431
MCF7
M
6
4
2
0
−2
174451100
174451200
174451300
174451400
174451500
position (bp)
RegionID: 30682, chr4:174451141−174451564−Beta_values
A431
MCF7
Beta
1.00
0.75
0.50
0.25
0.00
174451100
174451200
174451300
174451400
174451500
position (bp)

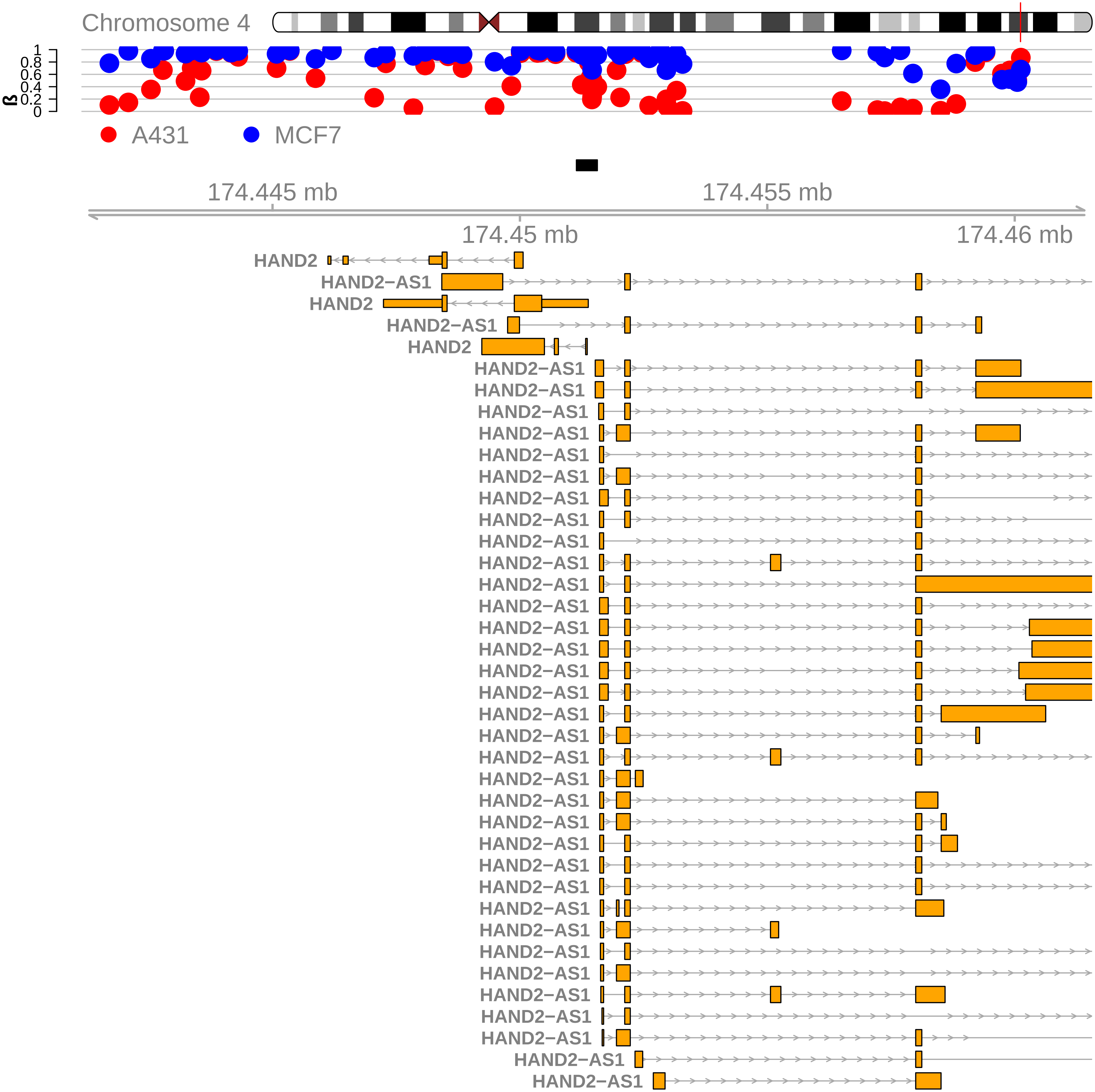

Chromosome 4
β
1
0.8
0.6
0.4
0.2
0
A431
MCF7
174.445 mb
174.455 mb
174.45 mb
174.46 mb
HAND2
HAND2−AS1
HAND2
HAND2−AS1
HAND2
HAND2−AS1
